# Short-Term Soy Protein Isolate Feeding Prevents Liver Steatosis and Reduces Serum ALT and AST Levels in Obese Female Zucker Rats

**DOI:** 10.3390/biomedicines6020055

**Published:** 2018-05-14

**Authors:** Reza Hakkak, C. Heath Gauss, Andrea Bell, Soheila Korourian

**Affiliations:** 1 Department of Dietetics and Nutrition, University of Arkansas for Medical Sciences, 4301 W. Markham St., Little Rock, AR 72205, USA; BellAC@archildrens.org; 2 Department of Pediatrics, University of Arkansas for Medical Sciences, Arkansas Children’s Hospital, 1 Children’s Way, Little Rock, AR 72202, USA; 3 Arkansas Children’s Research Institute, 13 Children’s Way, Little Rock, AR 72202, USA; 4 Department of Biostatistics, University of Arkansas for Medical Sciences, 4301 W. Markham St., Little Rock, AR 72205, USA; GaussClintonH@uams.edu; 5 Department of Pathology, University of Arkansas for Medical Sciences, 4301 W. Markham St., Little Rock, AR 72205, USA; Korouriansoheila@uams.edu

**Keywords:** obesity, non-alcoholic fatty liver disease, soy protein isolate

## Abstract

Non-alcoholic fatty liver disease is a common liver disorder worldwide and is associated with obesity. We investigated effects of obesity and short-term intake of soy protein with isoflavones (SPI) on body weight change, energy intake, liver steatosis, and serum aspartate aminotransferase (AST), alanine aminotransferase (ALT), and leptin levels. Seventeen lean and seventeen obese (*fa*/*fa*) female Zucker rats were randomly assigned to either casein or SPI diet for 8 weeks. Body weight was recorded twice weekly; feed intake was measured weekly. Livers were examined histologically, and serum AST, ALT, and leptin levels were measured. Obese soy-fed (OS) rats gained more weight but had lower liver steatosis than obese casein-fed (OC) rats. Energy intake for OS versus OC rats were only different at weeks 2 and 3. Serum AST and ALT levels were lower in OS versus OC rats. Obesity increased serum leptin levels for both diets. In summary, short-term SPI intake reduced liver steatosis, and the only time points at which the mean energy intakes of OS and OC rats differed were at weeks 2 and 3, where OS rats had a higher mean energy intake, which may have accounted for the increased body weight in OS rats.

## 1. Introduction

Obesity has been an epidemic in the United States (US) for more than three decades, and the rate of adult obesity continues to grow. Recent data from the Centers for Disease Control and Prevention (CDC, Atlanta, GA, USA) indicated that more than one-third of US adults are obese [1,2]. Worldwide, more than 1.9 billion adults are overweight, and over 600 million adults are obese [3,4]. Obesity is associated with serious health conditions including type 2 diabetes, cardiovascular disease, certain types of cancers, hyperlipidemia, and liver steatosis [5]. Non-alcoholic fatty liver disease (NAFLD), the major cause of abnormal liver function in the US and the world, is often associated with obesity. Mortality in individuals with NAFLD is significantly higher than in the general population, with liver-related complications being a common cause of death. An estimated 70 million adults have NAFLD. In obese individuals, fat accumulation in the abdominal region affects both lipid and glucose metabolism, and individuals with fatty liver (liver steatosis) are more likely to develop insulin-resistant diabetes. NAFLD is one of the common causes of liver diseases with a prevalence of up to 34% in the US [6,7], but this prevalence increases to more than 50% in the obese population.

It has been reported that soy protein intake can reduce serum cholesterol and triglycerides and also reduce accumulation of cholesterol and triglycerides in the liver which can lead to reduction of liver steatosis [8,9,10,11]. A study reported that soy protein with high isoflavones reduced liver lipids which resulted in reduction of cholesterol [12]. Another study showed that adding soy isoflavones to a casein diet lowered hepatic concentration of triglycerols compared to the casein diet. They reported decreased plasma aspartate aminotransferase (AST) and alanine aminotransferase (ALT) levels were accompanied with lower circulating alkaline phosphatase, bile acids; decreased plasma and liver mRNA levels of tumor necrosis factor- α (TNF-α), lower interleukin-1 β, and monocyte chemoattractant protein-1; and an increased anti-inflammatory fatty acid index in plasma [13]. Several mechanisms may be involved in the development and progress of liver steatosis [12,14]. Some studies have indicated that isoflavones in soy protein are responsible, in part, for the reduction of liver steatosis.

Some studies have used obese-diabetic Zucker rats fed a soy protein diet long-term and shown that a soy diet decreased serum and hepatic triglycerides and cholesterol concentrations that resulted in reductions in sterol regulatory element-binding transcription factor-1 (SREBP-1) expression. Also, soy protein increases hepatic fatty acid oxidation through Peroxisome proliferator-activated receptor-α (PPAR-α) [15]. In the same animal model, it was shown that rats fed soy diet had smaller and increased adipocytes than casein-fed rats, indicating that soy protein prevents hypertrophy and stimulates adipocyte hyperplasia with higher expression of PPAR-γ, which leads to an increased release of fatty acids into circulation [16]. Soy protein intake can have a hypolipidemic effect by reducing serum concentration of very-low-density lipoprotein (VLDL) triglycerides and LDL cholesterol in diabetic rats [17].

Previously, we reported that long-term (16 weeks) soy protein with high isoflavone (SPI) intake can reduce liver steatosis in an obese Zucker rat model [18,19] and that obese soy-fed (OS) rats gained more weight than obese casein-fed (OC) rats. The main objective of the present study was to investigate the effects of obesity and short-term (8 weeks) SPI consumption on body weight, energy intake, liver steatosis, and serum AST, ALT, and leptin levels in female Zucker rats.

## 2. Materials and Methods

### 2.1. Experimental Design

All animal care and procedures were approved by the University of Arkansas for Medical Sciences/Arkansas Children’s Research Institute Institutional Animal Care and Use Committee and adhered to the institutional policies and procedures. The guidelines of the United States Department of Agriculture (USDA, Washington, DC, USA) Animal Welfare Act were followed to ensure that the care and use of animals were appropriate and humane. A total of 34 five-week-old female lean and obese (*fa*/*fa*) rats were purchased from Harlan Industries (Indianapolis, IN, USA). Rats were housed in an Association-for-Assessment-and-Accreditation-of-Laboratory-Animal-Care-approved animal facility that is registered with the USDA and has a fully approved Letter of Assurance on file with the Office of Laboratory Animal Welfare of the National Institutes of Health. Rats were housed one per cage in 12-h light-dark cycles and had *ad libitum* access to feed and water. At the end of the 8-week experiment, rats were anesthetized using carbon dioxide and then euthanized by decapitation, and livers and serums were collected.

After one week of acclimation on an AIN-93G diet (American Institute of Nutrition), lean and obese rats (8–9/group) were randomly assigned to a semi-purified AIN-93G diet with protein sources of either casein (CAS, Columbus, OH, USA ) as control or soy protein isolate (SPI) for 8 weeks. Each diet contained 3.8 kcal/g of feed, and the percentage of kilocalories from macronutrients was as follows: protein 18.8%, carbohydrates 64%, and fat 17.2%. We used the same diet composition as previously reported [18,19].

**Figure 1:**
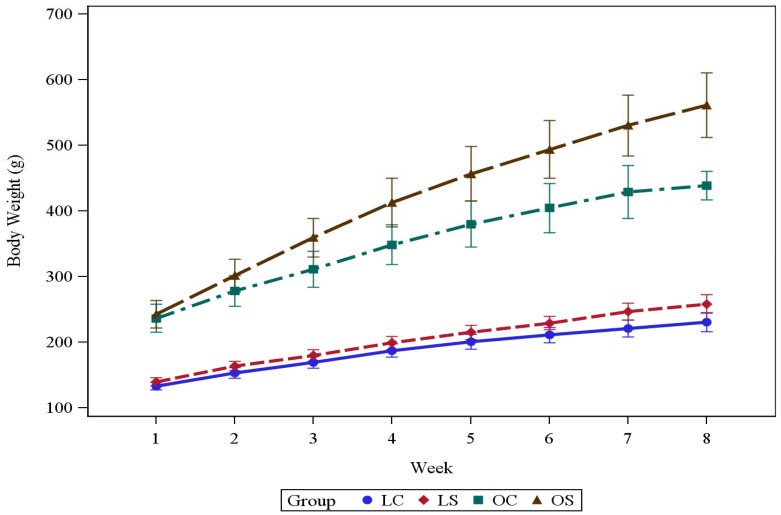
Body weight (g) across eight weeks for four groups (LC, LS, OC, and OS). LC = Lean Casein; LS = Lean Soy; OC = Obese Casein; and OS = Obese Soy. The mean ± SD is plotted in the figure at each week for each of the four body size/diet groups where each of the plotted means appears halfway between its corresponding set of error bars where the lower bar is one standard deviation below the mean and the upper bar is one standard deviation above the mean.

Rats were weighed twice weekly. Feed intake was measured once weekly and occurred over two days. The feed intake in each cage was measured and recorded in the morning on day one and then again in the morning on day two using a scale that rounded off to the nearest gram as we previously reported [20]. The average weekly body weight is shown in Figure 1.

### 2.2. Liver Histology and Weights

At the end of the experiment, livers were removed, and their weights were recorded. Liver weight was divided by final body weight to express liver weight as a percentage of body weight. Two 3-mm sections of each liver lobe were fixed in tissue cassettes and stored in 10% buffered formalin until histological examination. Upon examination, liver sections were fixed in 5% buffered formalin, cut, and stained with hematoxylin and eosin, and then they were examined by a pathologist in a blinded protocol. All of the liver sections were evaluated individually for the presence of microvesicular and macrovesicular steatosis blindly by a pathologist. The steatosis was semi-quantitated as a score of 0 to 4 in each case based on relative degree of steatosis within hepatocytes: (0) no steatosis; (1) <25%; (2) 25–50%; (3) >50–75% and (4) >75% as previously reported [21]. 

### 2.3. Serum Measurements

Levels of serum leptin were measured using rat enzyme-linked immunosorbent assay (ELISA) kits according to manufacturer’s protocol as previously reported [19]. Serum AST and ALT were measured using the VetScan VS2 profile. The analysis uses an immunoassay based on the measurement of enzyme mass in the serum [19].

### 2.4. Statistical Analysis

Descriptive statistics were obtained for variables of interest. Two-way analysis of variance with equal variances and two-way analysis of variance with unequal variances were utilized, as appropriate, for examining the end-of-experiment variables (absolute liver weight, liver weight expressed as a percentage of final body weight, steatosis scores, and serum leptin, AST, and ALT levels). For investigating change in body weight from the beginning (week 1) to the end of the experiment (week 8), analysis of covariance was employed. For analyzing body weight and energy intake across the eight time points of the experiment, repeated measures analyses were performed, which took into account the correlation among the repeated measurements for individual rats. Multiple comparisons were made using t-tests, with the Bonferroni method utilized for the multiple comparison adjustment. A *p*-value ≤ 0.05 was deemed significant. The statistical analyses were performed using SAS 9.4 (SAS Institute, Cary, NC, USA).

## 3. Results

### 3.1. Body Weight and Energy Intake

The body weights in grams (mean ± SD) at the beginning of the experiment were the following: lean casein (LC), 133 ± 5.6 g; lean soy (LS), 140 ± 6.1 g; obese casein (OC), 237 ± 21.1 g; and obese soy (OS), 243 ± 21.2 g. At the end of the 8-week experiment the body weights in grams (mean ± SD) were the following: LC, 231 ± 14.6 g; LS, 259 ± 14.4 g; OC, 439 ± 21.8 g; and OS, 561 ± 49.2 g. The mean body weight for each of the four groups of rats was greater at the end than at the beginning of the 8-week experiment (*p* < 0.0001) (Figure 1). The obese rats had a significantly different mean final body weight than lean rats regardless of their diets (*p* < 0.0001) with obese rats having a greater mean final body weight (95% CI for the difference for the SPI diet: (254.2, 351.6); 95% CI for the difference for the casein diet: (155.7, 259.0)). There was also a statistical difference between mean final body weights for OS rats and OC rats (*p* < 0.0001) where the OS rats weighed more on average (95% CI for the difference: (72.7, 173.2)); however, there was not sufficient statistical evidence to suggest that the mean final body weight for LS rats was significantly different from that of LC rats (*p* = 1.0000) (Table 1).

**Figure 2:**
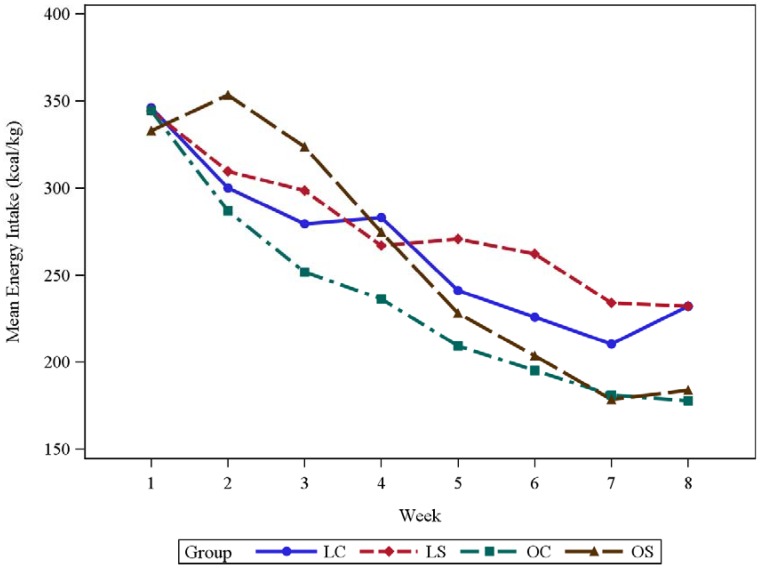
Mean energy intake (kcal/kg) across eight weeks for four body size/diet groups (LC, LS, OC, and OS). LC = Lean Casein; LS = Lean Soy; OC = Obese Casein; and OS = Obese Soy.

The weekly average energy intakes (kcal/kg) per body size/diet group are stated in Table 2 and graphically displayed in Figure 2, and *p*-values associated with certain statistical comparisons pertaining to energy intake are also presented in Table 2. The mean ± SD energy intakes at the beginning (week 1) of the experiment were the following: (1) LC, 346 ± 27.4 kcal/kg; (2) LS, 344 ± 23.1 kcal/kg; (3) OC, 344 ± 34.4 kcal/kg; and (4) OS, 333 ± 31.7 kcal/kg. The mean ± SD energy intakes for week 2 were the following: (1) LC, 300 ± 16.8 kcal/kg; (2) LS, 310 ± 18.3 kcal/kg; (3) OC, 287 ± 30.8 kcal/kg; and (4) OS, 353 ± 22.9 kcal/kg. For week 3, the mean ± SD energy intakes were (1) LC, 279 ± 30.5 kcal/kg; (2) LS, 298 ± 30.4 kcal/kg; (3) OC, 252 ± 19.1 kcal/kg; and (4) OS, 324 ± 15.6 kcal/kg. At weeks 2 and 3, the mean energy intake for OS rats was significantly different than that for OC rats (*p* < 0.0001) with OS rats having a higher mean energy intake (95% CI for the difference at week 2: (27.6, 105.4); 95% CI for the difference at week 3: (29.5, 114.2 kcal/kg)). At the end of the experiment, the energy intakes (mean ± SD) were the following: LC, 232 ± 25.8 kcal/kg; LS, 232 ± 30.5 kcal/kg; OC, 178 ± 18.2 kcal/kg; and OS, 184 ± 19.5 kcal/kg. There was not sufficient statistical evidence to suggest that the mean energy intakes for OC and OS rats at the beginning or end of the experiment were significantly different (*p* = 1.0000). However, at the end of the experiment, the mean energy intake for LC rats was significantly different than that for OC rats (*p* = 0.0034) with LC rats having a larger mean energy intake (95% CI for the difference: (11.8, 96.8)). Also, at the end of the experiment, the mean energy intake for LS rats was significantly different than that for OS rats (*p* = 0.0071) where the mean energy intake for LS rats was greater (95% CI for the difference: (8.2, 88.3)).

Summary statistics for liver weight expressed as absolute weight and for liver weight expressed as a percentage of final body weight are shown in Table 1. Mean absolute liver weights ± SD for the LC, LS, OC, and OS groups were 7.6 ± 0.9 g, 8.6 ± 0.8 g, 33.3 ± 4.4 g, and 20.2 ± 4.1 g, respectively. When liver weight was expressed as a percentage of final body weight for each group, the mean for the LC group was 3.3% ± 0.4%, 3.3% ± 0.3% for the LS group, 7.6% ± 0.7% for the OC group, and 3.6% ± 0.8% for the OS group. There was a statistical difference between the mean absolute liver weight for obese rats and that for lean rats for both diets (*p* < 0.0001) where the mean absolute liver weight was greater for obese rats (OC versus LC difference: 95% CI: (21.2, 30.2); OS versus LS difference: 95% CI: (7.6, 15.5)). For the CAS diet, there was a statistical difference between the mean liver weight as a percentage of final body weight for obese rats and that for lean rats (*p* < 0.0001) with obese rats having a greater mean (95% CI for the difference: (3.5, 5.1)), but there was not sufficient statistical evidence to suggest that there was a difference between obese and lean rats on the SPI diet (*p* = 1.0000). OS rats had a significantly different mean liver weight (expressed as absolute liver weight or as a percentage of final body weight) compared to OC rats (*p* < 0.0001) with the OS rats having a lower mean (95% CI for the difference in mean absolute liver weight: (−19.0, −7.3); 95% CI for the difference in mean percentage of final body weight: (−4.7, −3.2)).

**Figure 3:**
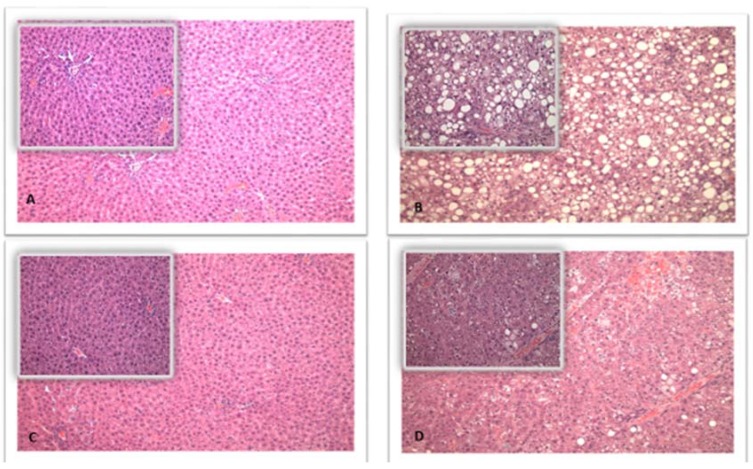
Liver Steatosis in lean (Casein (CAS) and soy protein with isoflavones (SPI)) and obese (CAS and SPI)-fed rats. Upper left liver (**A**) from a LC rat showing minimal steatosis, original magnification (100× and insert 200×); upper right liver (**B**) from an OC rat showing marked steatosis (>75% of hepatocytes exhibited microvesicular and macrovesicular steatosis), original magnification (100× and insert 200×); lower left liver (**C**) from a LS rat showing normal hepatic parenchyma with no steatosis, original magnification (100× and insert 200×); lower right liver (**D**) from an OS rat showing less steatosis (~25% of hepatocytes exhibited microvesicular and macrovesicular steatosis), original magnification (100× and insert 200×).

Representative photomicrographs of the hepatic parenchyma for lean and obese rats fed CAS or SPI diets are shown in Figure 3. The mean ± SD liver steatosis scores for the LC, LS, OC, and OS groups were 0.1 ± 0.4, 0.0 ± 0.0, 3.5 ± 0.5, and 1.2 ± 0.4, respectively. The mean liver steatosis score was significantly different for obese rats compared to lean rats for both CAS and SPI diets (*p* < 0.0001) with obese rats having a higher mean (OC versus LC difference: 95% CI: (2.9, 3.9); OS versus LS difference: 95% CI: (0.7, 1.7)). Comparing OS rats to OC rats, there was a significant difference between the mean liver steatosis scores (*p* < 0.0001) with the OS rats having a lower mean steatosis score (95% CI for the difference: (−2.8, −1.8).

### 3.2. Serum Measurement for Leptin, AST, and ALT

The mean ± SD serum leptin levels were 14.1 ± 5.3 ng/mL for the LC group, 20.0 ± 8.9 ng/mL for the LS group, 183.0 ± 22.3 ng/mL for the OC group, and 206.6 ± 25.7 ng/mL for the OS group. The mean serum leptin level was significantly different between obese rats and lean rats for both CAS and SPI diets (*p* < 0.0001) with obese rats having a higher mean level (OC versus LC difference: 95% CI: (146.4, 191.4); OS versus LS difference: 95% CI: (161.5, 211.9)). However, there was not sufficient statistical evidence to suggest that OS rats had a significantly different mean leptin level compared to OC rats (*p* = 0.2303) (Table 1).

Also, the mean serum levels of AST and ALT were significantly different for obese rats and lean rats that were on the CAS diet (*p* < 0.0001) with obese rats having a higher mean level (AST-OC versus LC difference: 95% CI: (79.1, 149.7); ALT-OC versus LC difference: 95% CI: (21.2, 45.8)), but there was not sufficient statistical evidence to suggest that there was a significant difference between obese and lean rats that were on the SPI diet (AST, *p* = 0.5834; ALT, *p* = 0.5823). Comparing OS and OC rats, the mean AST and ALT levels were significantly different (*p* < 0.0001 and *p* = 0.0357, respectively) with OS rats having lower mean levels (AST-OS versus OC difference: 95% CI: (−152.9, −85.4); ALT-OS versus OC difference: 95% CI: (−30.7, −0.8)) (Table 1).

## 4. Discussion

It has been known for long time that high energy intake in human and animal models will cause obesity which will lead in NAFLD development. There are several animal models to investigate the effects of high energy take on NAFLD development. For example, animals fed a high-fat diet mimicking both the histopathology and pathogenesis of human NAFLD present the same pathology features as in human NAFLD patients, including obesity. The Zucker rat (*fa*/*fa*) model is widely recognized as a rat model for obesity-related disease development. Obesity in the Zucker rat is inherited as an autosomal recessive trait caused by a mutation in the leptin receptor gene [22,23]. Animals homozygous for the *fa* allele become noticeably obese by 3 to 5 weeks of age, and by 14 weeks of age, their body composition is more than 40% lipid [24]. The most valuable contribution of the Zucker rat has been its utility as a model of human early-onset, hyperplastic-hypertrophic obesity. Many investigators have used this animal model to study the development, etiology, associated pathologies, possible treatments and contributing mechanisms of severe obesity [25]. In addition, this animal model develops hepatic steatosis due to dysregulated metabolic gene expression in the liver [10,26]. NAFLD is the major cause of abnormal liver function in the US and world. Data from our laboratory and from human studies suggest that obesity plays an important role in promotion of liver steatosis [18,19,20,21]. Previously, we reported that using the male and female obese Zucker rat model with long-term (16 weeks) consumption of diet containing SPI with high isoflavones lowered liver steatosis and caused a higher body weight compared to obese rats fed a casein diet [18,19]. However, the effect of short-term SPI intake on liver steatosis and body weight and energy intake is not clear. The major objectives of this study were to investigate the effects of obesity and short-term SPI consumption on (1) body weight and energy intake; (2) liver steatosis score; and (3) serum AST, ALT, and leptin levels. In the present study, we used female lean and obese Zucker rats placed on either a CAS- or SPI-diet for 8 weeks.

Soybeans and soy protein products are a major source of phytoestrogens, plant-based estrogen-like substances. Soybeans contain isoflavones which are structurally similar to mammalian estrogens and have estrogenic properties. The effects of soy isoflavones on human estrogen levels are complex. The soy isoflavones can bind to the estrogen receptor and induce estrogen-like effects in animals and humans [27]. The relative estrogenic potency is dependent on several factors such as animal species, route of administration, and dosage, duration, and timing of isoflavone exposure [27]. Phytoestrogens have relatively weak estrogen activity compared to animal estrogens; however, exposure to high dietary levels may result in biological responses in humans and animals, with favorable or unfavorable health consequences [28,29,30].

We found that obesity increased liver weight, steatosis score, and body weight for both CAS- and SPI-fed rats (*p* < 0.0001) and that OS rats had significantly lower liver steatosis and gained more weight than OC rats (*p* < 0.0001). Energy intakes for OS versus OC rats were only different at weeks 2 and 3 (*p* < 0.0001). Obesity caused a significant increase in AST and ALT levels for CAS-fed rats (*p* < 0.0001) but not SPI-fed rats (AST, *p* = 0.5834; ALT, *p* = 0.5823). There was a significant difference between OS and OC rats for AST level (*p* < 0.0001) and ALT level (*p* = 0.0357) with OS rats having lower AST and ALT levels. Obesity increased serum leptin level for both CAS- and soy-fed rats (*p* < 0.0001), but there was no difference between the OC and OS groups (*p* = 0.2303).

Several other studies are in agreement with this present data showing liver steatosis protection by an SPI diet. Results from our laboratory and others using an obese Zucker rat model [18,19,31] indicated that obese rats fed a long-term (16 weeks) SPI diet had significantly lower absolute liver weights [18,19,31] and lower liver weight as percentage of body weight [18,19] compared to obese rats fed diets of CAS [18,19,31], whey [31] or CAS plus arginine [19]. Also, livers from obese rats consuming a SPI diet had significantly less steatosis than livers of obese rats fed CAS- or whey-based diets [18,19,31]. Rodent research using an obese Zucker rat model has shown lighter liver eights [18,19,31,32] and decreased fat accumulation in the livers [18,19,32,33,34] of obese rats on a SPI diet compared to the livers of OC rats. Human studies report that SPI improved insulin sensitivity (a known risk factor for liver steatosis) [35] in postmenopausal women with abdominal obesity [36] and metabolic syndrome [37]. Some studies report the level or specific type of soy isoflavones may be responsible for lowering liver steatosis in obese rats. Peluso et al. [32] reported obese Zucker rats on low- and high-isoflavone diets for 70 days had 27% and 44% lighter livers, respectively, and 33% and 49% lower liver triglyceride concentrations, respectively, when compared to obese Zucker rats fed a casein diet for 70 days. Cain et al. [31] reported the livers of obese Zucker rats on a soy protein isolate diet with naturally occurring isoflavones were 40% lighter than the livers of obese Zucker rats on either a casein or whey-based diet. Recently, we investigated whether daidzein, a soy isoflavone in SPI, may be responsible for reduced liver steatosis in obese Zucker rats [20]. We used a casein-based diet with two different daidzein levels, a high-daidzein diet to match the amount of daidzein found in SPI with high isoflavones, and a low-daidzein diet to match the daidzein levels in low-isoflavone SPI. We found that there were no significant differences in mean liver weight, steatosis scores, body weights, energy intake, or serum leptin levels between diet groups. We concluded that daidzein may not be the main component of SPI responsible for increasing body weight or reducing liver steatosis in obese Zucker rats.

There is a strong, positive relationship between higher serum AST and ALT and NAFLD. It has been reported that obesity increases serum AST and ALT levels. Previously, we reported that long-term SPI feeding reduces both serum AST and ALT levels, a potential marker for reduction of liver steatosis [19]. In the present study, we investigated the effects of short-term SPI feeding on markers of liver function, such as serum AST and ALT, and found that obesity increased serum AST and ALT levels for CAS-fed rats (*p* < 0.0001) but not for SPI-fed rats. We also found that there was a significant difference between OS and OC rats for AST level (*p* < 0.0001) and ALT level (*p* = 0.0357) with OS rats having lower AST and ALT levels.

In the present study, we showed that OS compared to OC rats had a larger gain in body weight by the end of the 8-week experiment. Studies using male and female obese Zucker rats have shown that a diet containing SPI leads to increased weight gain compared to obese rats on a casein diet [18,19,31] or a low-isoflavone SPI diet [15]. Furthermore, lean Zucker rats also gain more weight on an SPI diet. There are two studies that reported that lean Zucker rats on diets containing SPI with high isoflavones (400 mg and 380 mg/kg of feed) gained significantly (*p* < 0.001, *p* < 0.0001) [31,38] more weight than lean Zucker rats on casein [23,30] or whey-based diets [31]. In comparison, a study by Peluso et al. [32] including male lean and obese Zucker rats and Sprague Dawley rats reported SPI with low (200 mg total isoflavones/kg of feed) and high isoflavones (2.89 g total isoflavones/kg of feed) did not significantly affect body weight in any of the rats compared to the casein-based control diet [32].

Regarding the effects of SPI on body weight, some studies [10,11,39] described how the experimental diets affected feed and kilocalorie intake. Peluso et al. [32] reported that lean and obese male Zucker rats on a SPI diet consumed 10% less kilocalories than the lean and obese rats on the casein-based control diet. Peluso et al. [32] also reported that male Sprague Dawley rats on the SPI diet consumed approximately 8% less kilocalories than those on the control diet. In contrast, in the present study, we reported that female obese Zucker rats fed a SPI diet gained significantly more weight than their casein-fed counterparts (*p* < 0.0001) and that OS rats consumed significantly more energy in week 2 and 3 than OC rats (*p* < 0.0001), which may have accounted for the higher weight gain seen in the SPI-fed group. This finding demonstrates that the effects of soy isoflavones on body weight and feed intake are highly variable. However, the most consistent effect of a diet containing SPI has been observed in obese Zucker rats. Several studies [13,15,18,19] reported that obese Zucker rats on SPI diets gained significantly more weight than OC rats (*p* < 0.05) and only one study [10] reported that obese Zucker rats on an SPI diet did not have significantly different weight gain from OC rats (*p* > 0.05). In the present study, we found that obesity the increased serum leptin levels for both CAS- and soy-fed rats, but there was no difference between the OC and OS groups.

## 5. Conclusions

In an obese Zucker rat model, we demonstrated that although obesity increases liver steatosis, a short-term (8 weeks) SPI-diet resulted in significantly decreased liver steatosis compared to CAS-fed rats despite higher body weight. Not only was liver steatosis lower in OS versus OC rats, but serum AST and ALT levels were also significantly lower in OS rats supporting liver pathology showing OS rats had healthier livers than OC rats. Furthermore, we concluded there were no differences in mean energy intake (kcal/kg) between the two diet groups at the beginning or end of the experiment. The OS group had a significantly higher mean energy intake at weeks 2 and 3 compared to the OC group, which may account for the increased mean body weight in OS rats. Further research is needed to understand the mechanisms enabling dietary SPI to protect against liver steatosis in the presence of obesity and, moreover, to interpret how this information translates to the context of human diets and human obesity.

## Figures and Tables

**Table 1:** Effects of obesity and diet containing casein or soy protein isolate on final body weight, liver weight, liver weight as % of body weight (BW), steatosis score, serum leptin (ng/mL), aspartate aminotransferase (AST) (U/L), and alanine aminotransferase (ALT) (U/L) (mean ± SD in the first 4 data columns) and statistical comparisons (*p*-values in the last 4 columns).

	LC	LS	OC	OS	LC vs. LS	LC vs. OC	LS vs. OS	OC vs. OS
Final BW (g)	231 ± 14.6	259 ± 14.4	439 ± 21.8	561 ± 49.2	1.0000	<0.0001	<0.0001	<0.0001
Liver weight (g)	7.6 ± 0.9	8.6 ± 0.8	33.3 ± 4.4	20.2 ± 4.1	0.0893	<0.0001	<0.0001	<0.0001
Liver weight (% BW)	3.3 ± 0.4	3.3 ± 0.3	7.6 ± 0.7	3.6 ± 0.8	1.0000	<0.0001	1.0000	<0.0001
Steatosis score	0.1 ± 0.4	0.0 ± 0.0	3.5 ± 0.5	1.2 ± 0.4	1.0000	<0.0001	<0.0001	<0.0001
Leptin	14.1 ± 5.3	20.0 ± 8.9	183.0 ± 22.3	206.6 ± 25.7	0.4524	<0.0001	<0.0001	0.2303
AST	108.1 ± 15.4	114.2 ± 17.4	222.5 ± 32.1	103.4 ± 12.1	1.0000	<0.0001	0.5834	<0.0001
ALT	38.4 ± 3.7	49.4 ± 8.9	71.9 ± 12.2	56.2 ± 10.2	0.0109	<0.0001	0.5823	0.0357

BW = body weight; LC = Lean Casein; LS = Lean Soy; OC = Obese Casein; and OS = Obese Soy. A *p*-value ≤ 0.05 was deemed statistically significant.

**Table 2:** Energy intake (kcal/kg) over eight weeks for four body size/diet groups (mean ± SD in the first 4 data columns) and statistical comparisons (*p*-values in the last 4 columns).

	LC	LS	OC	OS	LC vs. LS	LC vs. OC	LS vs. OS	OC vs. OS
Week 1	346 ± 27.4	344 ± 23.1	344 ± 34.4	333 ± 31.7	1.0000	1.0000	1.0000	1.0000
Week 2	300 ± 16.8	310 ± 18.3	287 ± 30.8	353 ± 22.9	1.0000	1.0000	0.0108	<0.0001
Week 3	279 ± 30.5	298 ± 30.4	252 ± 19.1	324 ± 15.6	1.0000	1.0000	1.0000	<0.0001
Week 4	283 ± 25.7	267 ± 37.7	236 ± 20.0	275 ± 20.3	1.0000	0.0611	1.0000	0.2459
Week 5	241 ± 31.5	271 ± 19.6	209 ± 34.7	228 ± 21.4	1.0000	0.9376	0.0828	1.0000
Week 6	226 ± 21.4	262 ± 34.1	195 ± 22.4	204 ± 18.6	0.2051	0.7391	0.0010	1.0000
Week 7	210 ± 28.4	234 ± 19.7	181 ± 20.3	179 ± 23.7	1.0000	0.6161	0.0007	1.0000
Week 8	232 ± 25.8	232 ± 30.5	178 ± 18.2	184 ± 19.5	1.0000	0.0034	0.0071	1.0000

LC = Lean Casein; LS = Lean Soy; OC = Obese Casein; and OS = Obese Soy. A *p*-value ≤ 0.05 was deemed statistically significant.3.2. Liver Weight and Steatosis Scores.
